# (*E*)-1-(1-Benzyl-5-methyl-1*H*-1,2,3-triazol-4-yl)-3-phenyl­prop-2-en-1-one

**DOI:** 10.1107/S1600536811037871

**Published:** 2011-09-30

**Authors:** Hoong-Kun Fun, Madhukar Hemamalini, Poovan Shanmugavelan, Alagusundaram Ponnuswamy, Rathinavel Jagatheesan

**Affiliations:** a X-ray Crystallography Unit, School of Physics, Universiti Sains Malaysia, 11800 USM, Penang, Malaysia; b Department of Organic Chemistry, School of Chemistry, Madurai Kamaraj University, Madurai-625 021, Tamil Nadu, India; c Department of Chemistry, Thanthai Hans Roever College, Perambalur-621 212, Tamil Nadu, India

## Abstract

The asymmetric unit of the title compound, C_19_H_17_N_3_O, contains two independent mol­ecules. In one mol­ecule, the essentially planar triazole ring [maximum deviation = 0.003 (2) Å] forms dihedral angles of 5.57 (12) and 87.51 (12)° with the two phenyl rings, while in the other mol­ecule [maximum deviation in triazole ring = 0.001 (2) Å] these angles are 1.55 (10) and 82.73 (11)°. The dihedral angles between the two phenyl rings in the two mol­ecules are 87.77 (13) and 81.22 (11)°. In the crystal, the independent mol­ecules are connected *via* a weak C—H⋯N hydrogen bond, forming dimers. Further stabilization is provided by weak C—H⋯π inter­actions.

## Related literature

For applications of 1,2,3-triazole compounds, see: Banerjee *et al.* (1996) ▶; Laliberte *et al.* (1967 ▶); Suwa *et al.* (1984 ▶). For applications of chalcones, see: Ballesteros *et al.* (1995 ▶); Kothari *et al.* (1999 ▶); Nagaraj & Reddy (2007 ▶).
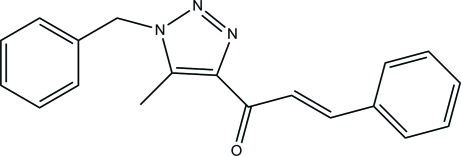


## Experimental

### 

#### Crystal data

C_19_H_17_N_3_O
*M* *_r_* = 303.36
Monoclinic, 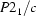

*a* = 12.3117 (14) Å
*b* = 13.8016 (15) Å
*c* = 19.312 (2) Å
β = 99.665 (2)°
*V* = 3235.0 (6) Å^3^
*Z* = 8
Mo *K*α radiation
μ = 0.08 mm^−1^
*T* = 296 K
0.46 × 0.33 × 0.11 mm

#### Data collection

Bruker APEXII DUO CCD area-detector diffractometer
Absorption correction: multi-scan (*SADABS*; Bruker, 2009 ▶) *T*_min_ = 0.965, *T*_max_ = 0.991
32508 measured reflections
9403 independent reflections
4890 reflections with *I* > 2σ(*I*)
*R*_int_ = 0.042

#### Refinement

*R*[*F* ^2^ > 2σ(*F* ^2^)] = 0.059
*wR*(*F* ^2^) = 0.219
*S* = 1.03
9403 reflections
417 parameters
H-atom parameters constrained
Δρ_max_ = 0.22 e Å^−3^
Δρ_min_ = −0.28 e Å^−3^

### 

Data collection: *APEX2* (Bruker, 2009 ▶); cell refinement: *SAINT* (Bruker, 2009 ▶); data reduction: *SAINT*; program(s) used to solve structure: *SHELXTL* (Sheldrick, 2008 ▶); program(s) used to refine structure: *SHELXTL*; molecular graphics: *SHELXTL*; software used to prepare material for publication: *SHELXTL* and *PLATON* (Spek, 2009 ▶).

## Supplementary Material

Crystal structure: contains datablock(s) global, I. DOI: 10.1107/S1600536811037871/lh5335sup1.cif

Structure factors: contains datablock(s) I. DOI: 10.1107/S1600536811037871/lh5335Isup2.hkl

Supplementary material file. DOI: 10.1107/S1600536811037871/lh5335Isup3.cml

Additional supplementary materials:  crystallographic information; 3D view; checkCIF report

## Figures and Tables

**Table 1:** Hydrogen-bond geometry (Å, °) *Cg*1 and *Cg*2 are the centroids of the C14*A*–C19*A* and C14*B*–C19*B* rings, respectively.

*D*—H⋯*A*	*D*—H	H⋯*A*	*D*⋯*A*	*D*—H⋯*A*
C13*A*—H13*A*⋯N1*B*^i^	0.97	2.50	3.453 (3)	166
C1*B*—H1*BA*⋯*Cg*1^ii^	0.93	2.97	3.893 (3)	174
C13*B*—H13*C*⋯*Cg*2^iii^	0.97	2.61	3.528 (2)	159

Symmetry codes: (i) 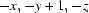; (ii) 
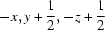
; (iii) 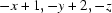.

**Fig. 1.**
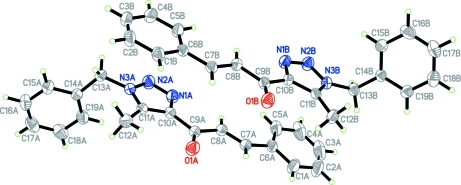
The asymmetric unit of the title compound showing 50% probability displacement ellipsoids.

**Fig. 2.**
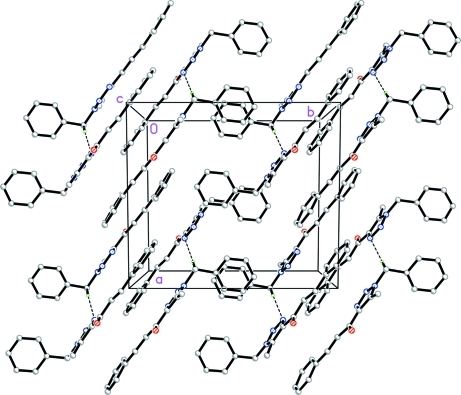
The crystal packing of the title compound (I). Hydrogen bonds are shown as dashed lines.

## supplementary crystallographic information

### Comment

Organic compounds, having the 1,2,3-triazole nucleus, may induce antiviral, agonist, antibacterial, antimicrobial, anti-HIV, anticonvulsants and anti-allergic properties.  In addition, compounds having 1,2,3-triazole group have found industrial application as dyes, corrosion inhibitors, sensors and photo-stabilizers (Banerjee *et al.*, 1996; Laliberte *et al.*, 1967; Suwa *et al.*, 1984).  The chalcone skeleton is a unique template for synthesizing various heterocyclic compounds.  The compounds with the backbone of chalcones were associated with different biological activities like cardiovascular, antispasmodic, anthelmintics, antiulcer, anti-inflammatory, antiviral, antiallergic, fungicidal, bactericidal, insecticidal, antitumor, herbicidal, anticancer, antitubercular and anti-HIV (Ballesteros *et al.*, 1995; Kothari *et al.*, 1999; Nagaraj & Reddy, 2007) properties.  Chalcones, considered as the precursors of flavonoids and isoflavonoids, are abundant in edible plants, and have also been shown to display a diverse array of pharmacological activities. The presence of a reactive α, β-unsaturated keto function in chalcones is found to be responsible for their activities.

The asymmetric unit of the title compound, (I), contains two crystallographically independent (*E*)-1-(1-benzyl-5-methyl-1*H*- 1,2,3-triazol-4-yl)-3-phenylprop-2-en-1-one molecules (A & B) as shown in Fig. 1. The triazole (N1A–N3A/C10A/C11A):(N1B–N3B/C10B/C11B) units are essentially planar, with maximum deviations of 0.003 (2) Å for atom C10A and  0.001 (2) Å for atom C11B. In molecule A the essentially planar triazole ring forms dihedral angles of 5.57 (12) and 87.51 (12)° with the two phenyl rings while in molecule B these angles are 1.55 (10) and 82.73 (11)°. The dihedral angles between the two phenyl(C1A–C6A/C14A–C19A): (C1B–C6B/C14B–C19B) rings in the independent molecules are 87.77 (13)° and 81.22 (11)° respectively.

In the crystal, (Fig. 2), two independent molecules are connected *via* intermolecular C—H···N hydrogen bonds (Table 1), forming dimers. Furthermore, the crystal structure is stabilized by weak C—H···π interactions involving the Cg1 (C14A–C19A) and Cg1 (C14B–C19B) rings.

### Experimental

A mixture of 4-acetyl-1-benzyl-5-methyl-1,2,3-triazole (0.20g, 0.93mmol) and benzaldehyde (0.98 g, 0.93 mmol) was stirred in ethanol (2–3 ml) and then 50% sodium hydroxide solution (0.5 ml) was added to it. The mixture was stirred for 3 minutes at room temperature and poured onto excess of crushed ice and neutralized with dilute hydrochloric acid. 1-Benzyl-5-methyl-1,2,3-triazol-4-yl-3-phenylprop-2-en-1-one precipitated as solid, which were filtered and recrystallized from ethanol. Yield: 0.27g (97%). M.p. 157–158°C.

### Refinement

All hydrogen atoms were positioned geometrically [C–H = 0.93–0.97 Å] and were refined using a riding model, with *U*_iso_(H) = 1.2 or 1.5 *U*_eq_(C).  A rotating group model was applied to the methyl groups.

### Crystal data

**Table:**

C_19_H_17_N_3_O	*F*(000) = 1280
*M**_r_* = 303.36	*D*_x_ = 1.246 Mg m^−^^3^
Monoclinic, *P*2_1_/*c*	Mo *K*α radiation, λ = 0.71073 Å
Hall symbol: -P 2ybc	Cell parameters from 5224 reflections
*a* = 12.3117 (14) Å	θ = 2.2–22.7°
*b* = 13.8016 (15) Å	µ = 0.08 mm^−^^1^
*c* = 19.312 (2) Å	*T* = 296 K
β = 99.665 (2)°	Block, colourless
*V* = 3235.0 (6) Å^3^	0.46 × 0.33 × 0.11 mm
*Z* = 8	

### Data collection

**Table:**

Bruker APEXII DUO CCD area-detector diffractometer	9403 independent reflections
Radiation source: fine-focus sealed tube	4890 reflections with *I* > 2σ(*I*)
graphite	*R*_int_ = 0.042
φ and ω scans	θ_max_ = 30.1°, θ_min_ = 1.7°
Absorption correction: multi-scan (*SADABS*; Bruker, 2009)	*h* = −16→17
*T*_min_ = 0.965, *T*_max_ = 0.991	*k* = −19→19
32508 measured reflections	*l* = −27→23

### Refinement

**Table:**

Refinement on *F*^2^	Primary atom site location: structure-invariant direct methods
Least-squares matrix: full	Secondary atom site location: difference Fourier map
*R*[*F*^2^ > 2σ(*F*^2^)] = 0.059	Hydrogen site location: inferred from neighbouring sites
*wR*(*F*^2^) = 0.219	H-atom parameters constrained
*S* = 1.03	*w* = 1/[σ^2^(*F*_o_^2^) + (0.1167*P*)^2^] where *P* = (*F*_o_^2^ + 2*F*_c_^2^)/3
9403 reflections	(Δ/σ)_max_ < 0.001
417 parameters	Δρ_max_ = 0.22 e Å^−^^3^
0 restraints	Δρ_min_ = −0.28 e Å^−^^3^

### Special details

**Table:**

Geometry. All s.u.'s (except the s.u. in the dihedral angle between two l.s. planes) are estimated using the full covariance matrix. The cell s.u.'s are taken into account individually in the estimation of s.u.'s in distances, angles and torsion angles; correlations between s.u.'s in cell parameters are only used when they are defined by crystal symmetry. An approximate (isotropic) treatment of cell s.u.'s is used for estimating s.u.'s involving l.s. planes.
Refinement. Refinement of F^2^ against ALL reflections. The weighted R-factor wR and goodness of fit S are based on F^2^, conventional R-factors R are based on F, with F set to zero for negative F^2^. The threshold expression of F^2^ > 2σ(F^2^) is used only for calculating R-factors(gt) etc. and is not relevant to the choice of reflections for refinement. R-factors based on F^2^ are statistically about twice as large as those based on F, and R- factors based on ALL data will be even larger.

### Fractional atomic coordinates and isotropic or equivalent isotropic displacement parameters (Å^2^)

**Table:**

	*x*	*y*	*z*	*U*_iso_*/*U*_eq_	
O1A	0.27709 (13)	0.39668 (11)	0.24684 (8)	0.0715 (4)	
N1A	0.16032 (15)	0.34511 (11)	0.06825 (9)	0.0608 (4)	
N2A	0.07387 (16)	0.29373 (13)	0.04346 (9)	0.0685 (5)	
N3A	0.03211 (14)	0.25962 (11)	0.09973 (9)	0.0565 (4)	
C1A	0.5850 (2)	0.60875 (17)	0.19015 (18)	0.0902 (8)	
H1AA	0.5916	0.6029	0.2387	0.108*	
C2A	0.6648 (3)	0.6594 (2)	0.1605 (3)	0.1247 (14)	
H2AA	0.7252	0.6859	0.1899	0.150*	
C3A	0.6558 (3)	0.6705 (2)	0.0899 (3)	0.1209 (15)	
H3AA	0.7094	0.7042	0.0711	0.145*	
C4A	0.5677 (3)	0.63209 (19)	0.04714 (19)	0.1042 (10)	
H4AA	0.5610	0.6400	−0.0012	0.125*	
C5A	0.4883 (2)	0.58161 (15)	0.07423 (14)	0.0766 (7)	
H5AA	0.4282	0.5565	0.0439	0.092*	
C6A	0.49611 (16)	0.56747 (12)	0.14621 (12)	0.0584 (5)	
C7A	0.41637 (16)	0.51129 (12)	0.17720 (11)	0.0545 (5)	
H7AA	0.4230	0.5142	0.2258	0.065*	
C8A	0.33539 (16)	0.45646 (12)	0.14430 (11)	0.0532 (5)	
H8AA	0.3236	0.4539	0.0955	0.064*	
C9A	0.26372 (16)	0.39958 (12)	0.18261 (10)	0.0505 (4)	
C10A	0.17414 (16)	0.34519 (12)	0.13959 (10)	0.0488 (4)	
C11A	0.09202 (15)	0.28976 (12)	0.16033 (10)	0.0490 (4)	
C12A	0.06483 (19)	0.26389 (17)	0.23003 (12)	0.0702 (6)	
H12A	−0.0134	0.2679	0.2283	0.105*	
H12B	0.1011	0.3080	0.2648	0.105*	
H12C	0.0892	0.1990	0.2420	0.105*	
C13A	−0.06699 (17)	0.20040 (14)	0.08836 (13)	0.0666 (6)	
H13A	−0.1071	0.2134	0.0416	0.080*	
H13B	−0.1136	0.2197	0.1218	0.080*	
C14A	−0.04614 (16)	0.09291 (13)	0.09558 (10)	0.0505 (4)	
C15A	−0.13275 (19)	0.03439 (16)	0.10419 (13)	0.0724 (6)	
H15A	−0.2003	0.0620	0.1082	0.087*	
C16A	−0.1209 (3)	−0.06517 (18)	0.10692 (16)	0.0901 (8)	
H16A	−0.1803	−0.1040	0.1129	0.108*	
C17A	−0.0221 (3)	−0.10682 (16)	0.10088 (13)	0.0848 (8)	
H17A	−0.0145	−0.1739	0.1016	0.102*	
C18A	0.0648 (3)	−0.04917 (17)	0.09374 (14)	0.0834 (7)	
H18A	0.1327	−0.0768	0.0909	0.100*	
C19A	0.0526 (2)	0.05009 (16)	0.09074 (14)	0.0730 (6)	
H19A	0.1125	0.0887	0.0853	0.088*	
O1B	0.23447 (13)	0.69469 (10)	0.24399 (7)	0.0659 (4)	
N1B	0.24505 (14)	0.73675 (12)	0.06272 (8)	0.0584 (4)	
N2B	0.31548 (15)	0.78740 (12)	0.03440 (8)	0.0624 (5)	
N3B	0.38931 (13)	0.82428 (10)	0.08821 (8)	0.0492 (4)	
C1B	−0.09734 (19)	0.47430 (14)	0.20382 (13)	0.0669 (6)	
H1BA	−0.0718	0.4804	0.2517	0.080*	
C2B	−0.1901 (2)	0.41806 (17)	0.18030 (19)	0.0877 (8)	
H2BA	−0.2260	0.3868	0.2126	0.105*	
C3B	−0.2287 (2)	0.40848 (16)	0.10989 (19)	0.0883 (8)	
H3BA	−0.2907	0.3708	0.0945	0.106*	
C4B	−0.1757 (2)	0.45471 (16)	0.06215 (15)	0.0787 (7)	
H4BA	−0.2016	0.4482	0.0143	0.094*	
C5B	−0.08397 (17)	0.51079 (14)	0.08503 (12)	0.0616 (5)	
H5BA	−0.0490	0.5422	0.0523	0.074*	
C6B	−0.04284 (15)	0.52120 (11)	0.15621 (10)	0.0489 (4)	
C7B	0.05448 (15)	0.57853 (12)	0.18235 (10)	0.0488 (4)	
H7BA	0.0790	0.5764	0.2306	0.059*	
C8B	0.11238 (15)	0.63355 (12)	0.14558 (10)	0.0496 (4)	
H8BA	0.0919	0.6371	0.0971	0.059*	
C9B	0.20855 (15)	0.68918 (12)	0.18018 (10)	0.0483 (4)	
C10B	0.27209 (14)	0.74073 (11)	0.13362 (9)	0.0442 (4)	
C11B	0.36537 (15)	0.79731 (11)	0.15077 (9)	0.0431 (4)	
C12B	0.43183 (18)	0.82731 (15)	0.21835 (10)	0.0625 (5)	
H12D	0.5082	0.8300	0.2137	0.094*	
H12E	0.4223	0.7813	0.2541	0.094*	
H12F	0.4081	0.8901	0.2312	0.094*	
C13B	0.47926 (17)	0.88434 (13)	0.07265 (11)	0.0550 (5)	
H13C	0.4861	0.8757	0.0237	0.066*	
H13D	0.5475	0.8627	0.1011	0.066*	
C14B	0.46353 (15)	0.99030 (12)	0.08633 (9)	0.0465 (4)	
C15B	0.36468 (17)	1.03723 (14)	0.06188 (12)	0.0635 (5)	
H15B	0.3053	1.0023	0.0381	0.076*	
C16B	0.3540 (2)	1.13550 (16)	0.07273 (14)	0.0780 (7)	
H16B	0.2875	1.1664	0.0567	0.094*	
C17B	0.4418 (2)	1.18741 (16)	0.10720 (13)	0.0772 (7)	
H17B	0.4345	1.2535	0.1145	0.093*	
C18B	0.5396 (2)	1.14258 (16)	0.13074 (12)	0.0731 (6)	
H18B	0.5991	1.1783	0.1535	0.088*	
C19B	0.55057 (17)	1.04482 (14)	0.12086 (10)	0.0582 (5)	
H19B	0.6174	1.0147	0.1376	0.070*	

### Atomic displacement parameters (Å^2^)

**Table:**

	*U*^11^	*U*^22^	*U*^33^	*U*^12^	*U*^13^	*U*^23^
O1A	0.0724 (10)	0.0830 (10)	0.0565 (9)	−0.0170 (8)	0.0029 (7)	0.0035 (7)
N1A	0.0682 (11)	0.0581 (9)	0.0556 (10)	−0.0100 (8)	0.0087 (8)	−0.0002 (7)
N2A	0.0774 (13)	0.0655 (10)	0.0592 (10)	−0.0148 (9)	0.0015 (9)	0.0014 (8)
N3A	0.0524 (9)	0.0460 (8)	0.0675 (11)	−0.0039 (7)	−0.0006 (8)	0.0069 (7)
C1A	0.0579 (14)	0.0708 (14)	0.134 (2)	−0.0107 (12)	−0.0069 (15)	−0.0123 (15)
C2A	0.0603 (17)	0.083 (2)	0.224 (5)	−0.0254 (15)	0.003 (2)	−0.006 (3)
C3A	0.089 (2)	0.0598 (15)	0.230 (5)	−0.0055 (16)	0.074 (3)	0.008 (2)
C4A	0.120 (3)	0.0661 (14)	0.142 (3)	−0.0103 (17)	0.067 (2)	0.0075 (16)
C5A	0.0793 (16)	0.0583 (12)	0.0964 (19)	−0.0121 (12)	0.0273 (14)	0.0028 (12)
C6A	0.0468 (10)	0.0385 (8)	0.0901 (16)	0.0027 (8)	0.0122 (10)	−0.0037 (9)
C7A	0.0493 (10)	0.0457 (9)	0.0668 (12)	0.0045 (8)	0.0045 (9)	−0.0013 (8)
C8A	0.0491 (10)	0.0495 (9)	0.0602 (12)	−0.0018 (8)	0.0066 (9)	−0.0004 (8)
C9A	0.0490 (10)	0.0447 (9)	0.0562 (12)	0.0017 (8)	0.0045 (9)	0.0029 (8)
C10A	0.0508 (10)	0.0416 (8)	0.0530 (11)	0.0018 (8)	0.0061 (8)	0.0029 (7)
C11A	0.0451 (10)	0.0400 (8)	0.0601 (11)	0.0036 (7)	0.0035 (8)	0.0058 (8)
C12A	0.0592 (13)	0.0796 (14)	0.0724 (14)	−0.0070 (11)	0.0125 (11)	0.0183 (11)
C13A	0.0484 (11)	0.0533 (10)	0.0912 (16)	−0.0041 (9)	−0.0082 (11)	0.0040 (10)
C14A	0.0484 (10)	0.0504 (9)	0.0512 (10)	−0.0041 (8)	0.0041 (8)	−0.0019 (8)
C15A	0.0571 (13)	0.0664 (13)	0.0930 (17)	−0.0101 (11)	0.0108 (12)	0.0058 (11)
C16A	0.091 (2)	0.0645 (13)	0.111 (2)	−0.0261 (14)	0.0064 (16)	0.0132 (13)
C17A	0.126 (2)	0.0488 (11)	0.0778 (16)	−0.0010 (15)	0.0108 (16)	−0.0007 (11)
C18A	0.100 (2)	0.0651 (13)	0.0914 (18)	0.0206 (14)	0.0343 (15)	0.0028 (12)
C19A	0.0648 (14)	0.0600 (11)	0.0987 (18)	0.0026 (11)	0.0266 (13)	0.0088 (11)
O1B	0.0717 (10)	0.0723 (9)	0.0529 (9)	−0.0186 (7)	0.0080 (7)	−0.0016 (7)
N1B	0.0594 (10)	0.0620 (9)	0.0519 (10)	−0.0128 (8)	0.0038 (8)	0.0007 (7)
N2B	0.0669 (11)	0.0679 (10)	0.0509 (10)	−0.0157 (9)	0.0057 (8)	−0.0004 (8)
N3B	0.0507 (9)	0.0444 (7)	0.0529 (9)	−0.0063 (7)	0.0099 (7)	−0.0007 (6)
C1B	0.0636 (13)	0.0617 (11)	0.0790 (15)	−0.0020 (10)	0.0224 (11)	0.0143 (10)
C2B	0.0636 (15)	0.0662 (14)	0.140 (3)	−0.0110 (12)	0.0360 (17)	0.0278 (15)
C3B	0.0551 (14)	0.0569 (12)	0.148 (3)	−0.0101 (11)	0.0024 (16)	0.0066 (15)
C4B	0.0667 (15)	0.0671 (13)	0.0966 (18)	−0.0092 (12)	−0.0031 (13)	−0.0088 (12)
C5B	0.0578 (12)	0.0557 (10)	0.0716 (14)	−0.0088 (10)	0.0116 (10)	−0.0009 (9)
C6B	0.0453 (9)	0.0379 (8)	0.0649 (12)	0.0017 (7)	0.0136 (9)	0.0016 (8)
C7B	0.0476 (10)	0.0432 (8)	0.0562 (10)	0.0022 (8)	0.0109 (8)	0.0009 (8)
C8B	0.0474 (10)	0.0480 (9)	0.0535 (11)	−0.0057 (8)	0.0092 (8)	−0.0008 (8)
C9B	0.0488 (10)	0.0419 (8)	0.0541 (11)	−0.0006 (8)	0.0082 (8)	−0.0021 (7)
C10B	0.0432 (9)	0.0388 (8)	0.0494 (10)	−0.0022 (7)	0.0040 (8)	−0.0014 (7)
C11B	0.0434 (9)	0.0356 (7)	0.0507 (10)	−0.0002 (7)	0.0093 (8)	−0.0002 (7)
C12B	0.0605 (12)	0.0674 (11)	0.0575 (12)	−0.0157 (10)	0.0035 (10)	−0.0029 (9)
C13B	0.0525 (11)	0.0504 (9)	0.0661 (12)	−0.0042 (8)	0.0219 (9)	0.0036 (8)
C14B	0.0454 (10)	0.0471 (9)	0.0493 (10)	−0.0045 (8)	0.0151 (8)	0.0044 (7)
C15B	0.0476 (11)	0.0606 (11)	0.0823 (15)	−0.0027 (9)	0.0108 (10)	0.0075 (10)
C16B	0.0713 (15)	0.0661 (13)	0.1014 (19)	0.0187 (12)	0.0285 (14)	0.0176 (13)
C17B	0.101 (2)	0.0516 (11)	0.0844 (16)	−0.0004 (13)	0.0319 (15)	−0.0062 (11)
C18B	0.0851 (17)	0.0654 (13)	0.0688 (14)	−0.0132 (13)	0.0127 (13)	−0.0163 (11)
C19B	0.0563 (12)	0.0641 (11)	0.0531 (11)	−0.0032 (9)	0.0063 (9)	−0.0002 (9)

### Geometric parameters (Å, °)

**Table:**

O1A—C9A	1.224 (2)	O1B—C9B	1.222 (2)
N1A—N2A	1.302 (2)	N1B—N2B	1.304 (2)
N1A—C10A	1.360 (2)	N1B—C10B	1.355 (2)
N2A—N3A	1.362 (2)	N2B—N3B	1.359 (2)
N3A—C11A	1.340 (2)	N3B—C11B	1.343 (2)
N3A—C13A	1.454 (3)	N3B—C13B	1.454 (2)
C1A—C6A	1.390 (3)	C1B—C6B	1.386 (3)
C1A—C2A	1.403 (5)	C1B—C2B	1.392 (3)
C1A—H1AA	0.9300	C1B—H1BA	0.9300
C2A—C3A	1.358 (5)	C2B—C3B	1.370 (4)
C2A—H2AA	0.9300	C2B—H2BA	0.9300
C3A—C4A	1.356 (5)	C3B—C4B	1.373 (4)
C3A—H3AA	0.9300	C3B—H3BA	0.9300
C4A—C5A	1.374 (4)	C4B—C5B	1.379 (3)
C4A—H4AA	0.9300	C4B—H4BA	0.9300
C5A—C6A	1.391 (3)	C5B—C6B	1.390 (3)
C5A—H5AA	0.9300	C5B—H5BA	0.9300
C6A—C7A	1.456 (3)	C6B—C7B	1.454 (3)
C7A—C8A	1.326 (3)	C7B—C8B	1.326 (3)
C7A—H7AA	0.9300	C7B—H7BA	0.9300
C8A—C9A	1.470 (3)	C8B—C9B	1.474 (3)
C8A—H8AA	0.9300	C8B—H8BA	0.9300
C9A—C10A	1.471 (3)	C9B—C10B	1.471 (3)
C10A—C11A	1.380 (3)	C10B—C11B	1.382 (2)
C11A—C12A	1.484 (3)	C11B—C12B	1.478 (3)
C12A—H12A	0.9600	C12B—H12D	0.9600
C12A—H12B	0.9600	C12B—H12E	0.9600
C12A—H12C	0.9600	C12B—H12F	0.9600
C13A—C14A	1.508 (3)	C13B—C14B	1.504 (2)
C13A—H13A	0.9700	C13B—H13C	0.9700
C13A—H13B	0.9700	C13B—H13D	0.9700
C14A—C19A	1.369 (3)	C14B—C19B	1.385 (3)
C14A—C15A	1.370 (3)	C14B—C15B	1.389 (3)
C15A—C16A	1.382 (3)	C15B—C16B	1.382 (3)
C15A—H15A	0.9300	C15B—H15B	0.9300
C16A—C17A	1.368 (4)	C16B—C17B	1.372 (4)
C16A—H16A	0.9300	C16B—H16B	0.9300
C17A—C18A	1.359 (4)	C17B—C18B	1.362 (3)
C17A—H17A	0.9300	C17B—H17B	0.9300
C18A—C19A	1.378 (3)	C18B—C19B	1.372 (3)
C18A—H18A	0.9300	C18B—H18B	0.9300
C19A—H19A	0.9300	C19B—H19B	0.9300
			
N2A—N1A—C10A	109.19 (16)	N2B—N1B—C10B	109.54 (15)
N1A—N2A—N3A	106.85 (16)	N1B—N2B—N3B	106.65 (15)
C11A—N3A—N2A	111.31 (16)	C11B—N3B—N2B	111.36 (15)
C11A—N3A—C13A	129.11 (18)	C11B—N3B—C13B	129.29 (16)
N2A—N3A—C13A	119.57 (17)	N2B—N3B—C13B	119.34 (15)
C6A—C1A—C2A	119.3 (3)	C6B—C1B—C2B	120.4 (2)
C6A—C1A—H1AA	120.4	C6B—C1B—H1BA	119.8
C2A—C1A—H1AA	120.4	C2B—C1B—H1BA	119.8
C3A—C2A—C1A	121.3 (3)	C3B—C2B—C1B	120.5 (2)
C3A—C2A—H2AA	119.3	C3B—C2B—H2BA	119.8
C1A—C2A—H2AA	119.3	C1B—C2B—H2BA	119.8
C4A—C3A—C2A	119.4 (3)	C2B—C3B—C4B	119.8 (2)
C4A—C3A—H3AA	120.3	C2B—C3B—H3BA	120.1
C2A—C3A—H3AA	120.3	C4B—C3B—H3BA	120.1
C3A—C4A—C5A	120.9 (3)	C3B—C4B—C5B	120.1 (3)
C3A—C4A—H4AA	119.6	C3B—C4B—H4BA	119.9
C5A—C4A—H4AA	119.6	C5B—C4B—H4BA	119.9
C4A—C5A—C6A	121.2 (3)	C4B—C5B—C6B	121.2 (2)
C4A—C5A—H5AA	119.4	C4B—C5B—H5BA	119.4
C6A—C5A—H5AA	119.4	C6B—C5B—H5BA	119.4
C1A—C6A—C5A	117.9 (2)	C1B—C6B—C5B	118.10 (19)
C1A—C6A—C7A	118.9 (2)	C1B—C6B—C7B	119.10 (19)
C5A—C6A—C7A	123.2 (2)	C5B—C6B—C7B	122.80 (17)
C8A—C7A—C6A	127.7 (2)	C8B—C7B—C6B	127.70 (19)
C8A—C7A—H7AA	116.1	C8B—C7B—H7BA	116.2
C6A—C7A—H7AA	116.1	C6B—C7B—H7BA	116.2
C7A—C8A—C9A	122.02 (19)	C7B—C8B—C9B	121.24 (18)
C7A—C8A—H8AA	119.0	C7B—C8B—H8BA	119.4
C9A—C8A—H8AA	119.0	C9B—C8B—H8BA	119.4
O1A—C9A—C8A	122.50 (17)	O1B—C9B—C10B	120.71 (16)
O1A—C9A—C10A	121.08 (17)	O1B—C9B—C8B	122.92 (17)
C8A—C9A—C10A	116.41 (17)	C10B—C9B—C8B	116.36 (16)
N1A—C10A—C11A	108.70 (16)	N1B—C10B—C11B	108.57 (15)
N1A—C10A—C9A	121.82 (17)	N1B—C10B—C9B	122.18 (16)
C11A—C10A—C9A	129.47 (18)	C11B—C10B—C9B	129.23 (16)
N3A—C11A—C10A	103.95 (17)	N3B—C11B—C10B	103.88 (15)
N3A—C11A—C12A	122.85 (18)	N3B—C11B—C12B	122.95 (16)
C10A—C11A—C12A	133.19 (18)	C10B—C11B—C12B	133.17 (17)
C11A—C12A—H12A	109.5	C11B—C12B—H12D	109.5
C11A—C12A—H12B	109.5	C11B—C12B—H12E	109.5
H12A—C12A—H12B	109.5	H12D—C12B—H12E	109.5
C11A—C12A—H12C	109.5	C11B—C12B—H12F	109.5
H12A—C12A—H12C	109.5	H12D—C12B—H12F	109.5
H12B—C12A—H12C	109.5	H12E—C12B—H12F	109.5
N3A—C13A—C14A	114.42 (17)	N3B—C13B—C14B	113.13 (15)
N3A—C13A—H13A	108.7	N3B—C13B—H13C	109.0
C14A—C13A—H13A	108.7	C14B—C13B—H13C	109.0
N3A—C13A—H13B	108.7	N3B—C13B—H13D	109.0
C14A—C13A—H13B	108.7	C14B—C13B—H13D	109.0
H13A—C13A—H13B	107.6	H13C—C13B—H13D	107.8
C19A—C14A—C15A	118.16 (18)	C19B—C14B—C15B	118.23 (17)
C19A—C14A—C13A	123.90 (18)	C19B—C14B—C13B	120.04 (17)
C15A—C14A—C13A	117.86 (18)	C15B—C14B—C13B	121.65 (17)
C14A—C15A—C16A	120.8 (2)	C16B—C15B—C14B	120.4 (2)
C14A—C15A—H15A	119.6	C16B—C15B—H15B	119.8
C16A—C15A—H15A	119.6	C14B—C15B—H15B	119.8
C17A—C16A—C15A	120.3 (2)	C17B—C16B—C15B	119.9 (2)
C17A—C16A—H16A	119.9	C17B—C16B—H16B	120.0
C15A—C16A—H16A	119.9	C15B—C16B—H16B	120.0
C18A—C17A—C16A	119.3 (2)	C18B—C17B—C16B	120.3 (2)
C18A—C17A—H17A	120.4	C18B—C17B—H17B	119.8
C16A—C17A—H17A	120.4	C16B—C17B—H17B	119.8
C17A—C18A—C19A	120.3 (2)	C17B—C18B—C19B	120.1 (2)
C17A—C18A—H18A	119.9	C17B—C18B—H18B	119.9
C19A—C18A—H18A	119.9	C19B—C18B—H18B	119.9
C14A—C19A—C18A	121.2 (2)	C18B—C19B—C14B	121.0 (2)
C14A—C19A—H19A	119.4	C18B—C19B—H19B	119.5
C18A—C19A—H19A	119.4	C14B—C19B—H19B	119.5
			
C10A—N1A—N2A—N3A	−0.5 (2)	C10B—N1B—N2B—N3B	0.2 (2)
N1A—N2A—N3A—C11A	0.2 (2)	N1B—N2B—N3B—C11B	−0.3 (2)
N1A—N2A—N3A—C13A	178.98 (16)	N1B—N2B—N3B—C13B	179.68 (15)
C6A—C1A—C2A—C3A	1.3 (5)	C6B—C1B—C2B—C3B	−0.1 (3)
C1A—C2A—C3A—C4A	0.1 (5)	C1B—C2B—C3B—C4B	0.0 (4)
C2A—C3A—C4A—C5A	−0.5 (5)	C2B—C3B—C4B—C5B	−0.2 (4)
C3A—C4A—C5A—C6A	−0.6 (4)	C3B—C4B—C5B—C6B	0.5 (3)
C2A—C1A—C6A—C5A	−2.4 (3)	C2B—C1B—C6B—C5B	0.4 (3)
C2A—C1A—C6A—C7A	177.2 (2)	C2B—C1B—C6B—C7B	−179.32 (18)
C4A—C5A—C6A—C1A	2.1 (3)	C4B—C5B—C6B—C1B	−0.6 (3)
C4A—C5A—C6A—C7A	−177.5 (2)	C4B—C5B—C6B—C7B	179.11 (18)
C1A—C6A—C7A—C8A	−170.40 (19)	C1B—C6B—C7B—C8B	−174.84 (18)
C5A—C6A—C7A—C8A	9.2 (3)	C5B—C6B—C7B—C8B	5.4 (3)
C6A—C7A—C8A—C9A	176.99 (17)	C6B—C7B—C8B—C9B	178.79 (16)
C7A—C8A—C9A—O1A	−2.8 (3)	C7B—C8B—C9B—O1B	−6.5 (3)
C7A—C8A—C9A—C10A	177.47 (16)	C7B—C8B—C9B—C10B	174.80 (16)
N2A—N1A—C10A—C11A	0.6 (2)	N2B—N1B—C10B—C11B	−0.1 (2)
N2A—N1A—C10A—C9A	−178.32 (16)	N2B—N1B—C10B—C9B	−179.03 (16)
O1A—C9A—C10A—N1A	−177.83 (18)	O1B—C9B—C10B—N1B	−179.47 (17)
C8A—C9A—C10A—N1A	1.9 (2)	C8B—C9B—C10B—N1B	−0.8 (2)
O1A—C9A—C10A—C11A	3.5 (3)	O1B—C9B—C10B—C11B	1.8 (3)
C8A—C9A—C10A—C11A	−176.79 (17)	C8B—C9B—C10B—C11B	−179.48 (16)
N2A—N3A—C11A—C10A	0.2 (2)	N2B—N3B—C11B—C10B	0.22 (19)
C13A—N3A—C11A—C10A	−178.45 (17)	C13B—N3B—C11B—C10B	−179.73 (16)
N2A—N3A—C11A—C12A	179.62 (17)	N2B—N3B—C11B—C12B	−179.80 (17)
C13A—N3A—C11A—C12A	1.0 (3)	C13B—N3B—C11B—C12B	0.3 (3)
N1A—C10A—C11A—N3A	−0.46 (19)	N1B—C10B—C11B—N3B	−0.09 (19)
C9A—C10A—C11A—N3A	178.38 (17)	C9B—C10B—C11B—N3B	178.77 (16)
N1A—C10A—C11A—C12A	−179.8 (2)	N1B—C10B—C11B—C12B	179.93 (19)
C9A—C10A—C11A—C12A	−1.0 (3)	C9B—C10B—C11B—C12B	−1.2 (3)
C11A—N3A—C13A—C14A	−82.2 (3)	C11B—N3B—C13B—C14B	−74.4 (2)
N2A—N3A—C13A—C14A	99.3 (2)	N2B—N3B—C13B—C14B	105.67 (19)
N3A—C13A—C14A—C19A	−20.1 (3)	N3B—C13B—C14B—C19B	135.50 (18)
N3A—C13A—C14A—C15A	163.1 (2)	N3B—C13B—C14B—C15B	−48.0 (3)
C19A—C14A—C15A—C16A	−0.9 (3)	C19B—C14B—C15B—C16B	−0.7 (3)
C13A—C14A—C15A—C16A	176.1 (2)	C13B—C14B—C15B—C16B	−177.32 (19)
C14A—C15A—C16A—C17A	−0.2 (4)	C14B—C15B—C16B—C17B	0.7 (3)
C15A—C16A—C17A—C18A	1.5 (4)	C15B—C16B—C17B—C18B	0.1 (4)
C16A—C17A—C18A—C19A	−1.7 (4)	C16B—C17B—C18B—C19B	−0.8 (4)
C15A—C14A—C19A—C18A	0.6 (4)	C17B—C18B—C19B—C14B	0.7 (3)
C13A—C14A—C19A—C18A	−176.2 (2)	C15B—C14B—C19B—C18B	0.0 (3)
C17A—C18A—C19A—C14A	0.7 (4)	C13B—C14B—C19B—C18B	176.67 (19)

### Hydrogen-bond geometry (Å, °)

**Table:**

Cg1 and Cg2 are the centroids of the C14A–C19A and C14B–C19B rings, respectively.

**Table:**

*D*—H···*A*	*D*—H	H···*A*	*D*···*A*	*D*—H···*A*
C13A—H13A···N1B^i^	0.97	2.50	3.453 (3)	166
C1B—H1BA···Cg1^ii^	0.93	2.97	3.893 (3)	174
C13B—H13C···Cg2^iii^	0.97	2.61	3.528 (2)	159

Symmetry codes: (i) −*x*, −*y*+1, −*z*; (ii) −*x*, *y*+1/2, −*z*+1/2; (iii) −*x*+1, −*y*+2, −*z*.
